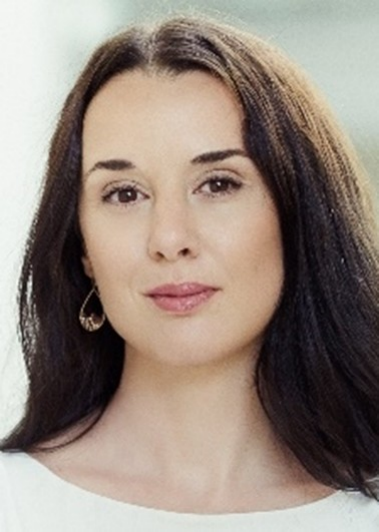# MALDI Matrices for the Analysis of Low Molecular Weight Compounds: Rational Design, Challenges and Perspectives

**DOI:** 10.1002/asia.202100044

**Published:** 2021-03-19

**Authors:** Zhi Qiao, Franziska Lissel

**Affiliations:** ^1^ Institute of Macromolecular Chemistry, Leibniz Institute for Polymer Research Dresden, Hohe Str. 6, 01069 Dresden (Germany) Faculty of Chemistry and Food Chemistry Dresden University of Technology, Mommsenstr. 4 01062 Dresden Germany; ^2^ Institute of Organic Chemistry and Macromolecular Chemistry Friedrich Schiller University Jena Humboldtstr. 10 07743 Jena Germany

**Keywords:** MALDI MS, MSI, matrix, LMW compounds

## Abstract

The analysis of low molecular weight (LMW) compounds is of great interest to detect small pharmaceutical drugs rapidly and sensitively, or to trace and understand metabolic pathways. Matrix‐assisted laser desorption/ionization mass spectrometry (MALDI MS) plays a central role in the analysis of high molecular weight (bio)molecules. However, its application for LMW compounds is restricted by spectral interferences in the low m/z region, which are produced by conventional organic matrices. Several strategies regarding sample preparation have been investigated to overcome this problem. A different rationale is centred on developing new matrices which not only meet the fundamental requirements of good absorption and high ionization efficiency, but are also vacuum stable and “MALDI silent”, i. e., do not give matrix‐related signals in the LMW area. This review gives an overview on the rational design strategies used to develop matrix systems for the analysis of LMW compounds, focusing on (i) the modification of well‐known matrices, (ii) the search for high molecular weight matrices, (iii) the development of binary, hybrid and nanomaterial‐based matrices, (iv) the advance of reactive matrices and (v) the progress made regarding matrices for negative or dual polarity mode.

## Graphical abstract

**To use MALDI MS and MS imaging** for the analysis of low molecular weight (LMW) analytes, new matrix systems are needed which combine suitable absorption and high ionization efficiency in dual polarity mode with being vacuum stable and “MALDI silent”, i.e., enabling noise‐free measurements in the LMW area. This Minireview summarizes recent research regarding the design and synthesis of such matrices, and discusses recurring design strategies and motifs.

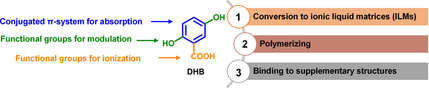

## 1 Introduction

Matrix‐assisted laser desorption/ionization mass spectrometry (MALDI MS)[^1^, ^2^] enables the rapid analysis of high molecular weight (HMW) biomolecules (e. g. proteins, polysaccharides etc.) and polymers, has a high detection accuracy and sensitivity, and usually yields single charged ions.[^3^, ^4^, ^5^, ^6^, ^7^] The matrix is central to the MALDI process, and as analyte incorporation as well as the desorption and ionization efficiency can differ between given matrix/analyte pairs, choosing the right matrix is a key success driver in MALDI MS.^[8]^ In the past decades, various matrices suitable for different macromolecule classes have been developed: For example, *α*‐cyano‐4‐hydroxycinnamic acid (CHCA) and 2,5‐dihydroxybenzoic acid (DHB) are excellent and ubiquitously used matrices for proteins, peptides and polysaccharides,[^9^, ^10^, ^11^] while *trans‐*2‐[3‐(4‐*t*‐butyl‐phenyl)‐2‐methyl‐2‐propenylidene]malononitrile (DCTB) has a high ionization efficiency for conjugated polymers.^[12]^

While MALDI MS is a much relied‐on standard tool for HMW analytics, LMW compounds (with m/z < 1000) – which cover a wide range of metabolite classes, e. g., amino acids, hormones, saccharides etc., are usually investigated with other MS methods.[^13^, ^14^, ^15^] But especially with the advance of MS imaging (MSI) techniques, there is now a significant interest to make LMW compounds analytics accessible with MALDI MS. MALDI‐MSI is a simple, rapid and sensitive method to visualize the spatial distribution of interesting molecules, e. g., small pharmaceutical drugs, on tissue sections[^16^, ^17^] and to trace metabolic pathways.^[4]^ Yet traditional MALDI matrices – small molecules with conjugated systems and acidic or basic functional groups ‐ usually do not support the analysis of LMW compounds^[18]^ as they desorb and ionize themselves, generating large amount of ion peaks in the LMW region.^[19]^ Still, conventional organic matrices can be used for the analysis of LMW compound classes, e. g., when the matrix‐related peaks do not overlap with the peaks of the analytical target. Finding a suitable matrix is often a tedious empirical process.[^20^, ^21^] Mapping and analyzing the influence of the chemical structure of the matrix on the matrix performance could allow to predict promising structures for targeted LMW analytics. Yet such systematic studies are rare. A notable exception is the systematic review of phenyl‐*α*‐cyano‐cinnamic acid derivatives carried out by the group of Junker:^[22]^ a chemical library of 59 derivatives was synthesized, and their performance for the analysis of lipids evaluated with two different laser systems.

This review aims to summarize work committed to the development of new matrix systems enabling MALDI MS and MSI for LMW analytes. The first section summarizes the general requirements for matrices suitable for LMW analytics and gives a short overview on strategies investigated to allow the utilization of classic small organic matrices (SOMs), such as sample pretreatments or instrument improvements. The second and longer section explores the rational design of new matrix systems, including (i) structural modifications of classic SOMs, (ii) the exploration of HMW matrices, (iii) the development of binary, hybrid and nanomaterial‐based matrices, (iv) the advance of reactive matrices and (v) the progress made regarding matrices for negative or dual polarity mode.

## 2 Utilizing classic matrices for LMW compound analytics

### 2.1 The MALDI process and general matrix requirements for LMW compound analytics

As implicated in the term “matrix‐assisted laser desorption/ionization”, the MALDI process centres on the desorption of the analyte, i. e., its transfer into the vapor phase, and the generation of charged analyte species, which are subsequently accelerated in an electric field and finally separated according to their m/z ratio.^[23]^ Briefly summarized, the energy of a pulsed laser (usually in the ultraviolet region) is absorbed by the matrix molecules, leading to their vaporization. The co‐crystallized analyte molecules are vaporized along with the matrix molecules and ionized via ion (e. g., H^+^) or charge transfer processes. The ionization process was intensively studied, and several mechanisms proposed[^22^, ^24^] (e. g., a two‐step mechanism differentiating primary (i. e. formation and separation of ions during desorption) and secondary (i. e. formation of secondary ions after the primary ionization) ionization, the “lucky survivor” model,[^25^, ^26^, ^27^] or the “coupled physical and chemical dynamics” (CPCD) model.^[28]^

Matrices suitable for LMW compound analytics must meet the same fundamental requirements as classic SOMs, i. e., (i) high absorption at the laser wavelength (e. g., Nd:YAG with a wavelength λ_laser_=355 nm), (ii) efficient ionization of the analyte via proton or electron transfer reactions, and (iii) homogenous layers to keep local variations to a minimum. In addition, a matrix for LMW compound analytics needs to be (iv) “MALDI silent”, i. e., do not cause matrix related ion peaks (background interferences) in the LMW area. For LMW compound analytics with MALDI MSI, the matrix furthermore has to be (v) vacuum stable, i. e., show a low sublimation rate in (ultra)high vacuum.[^21^, ^29^, ^30^]

**Figure 1:**
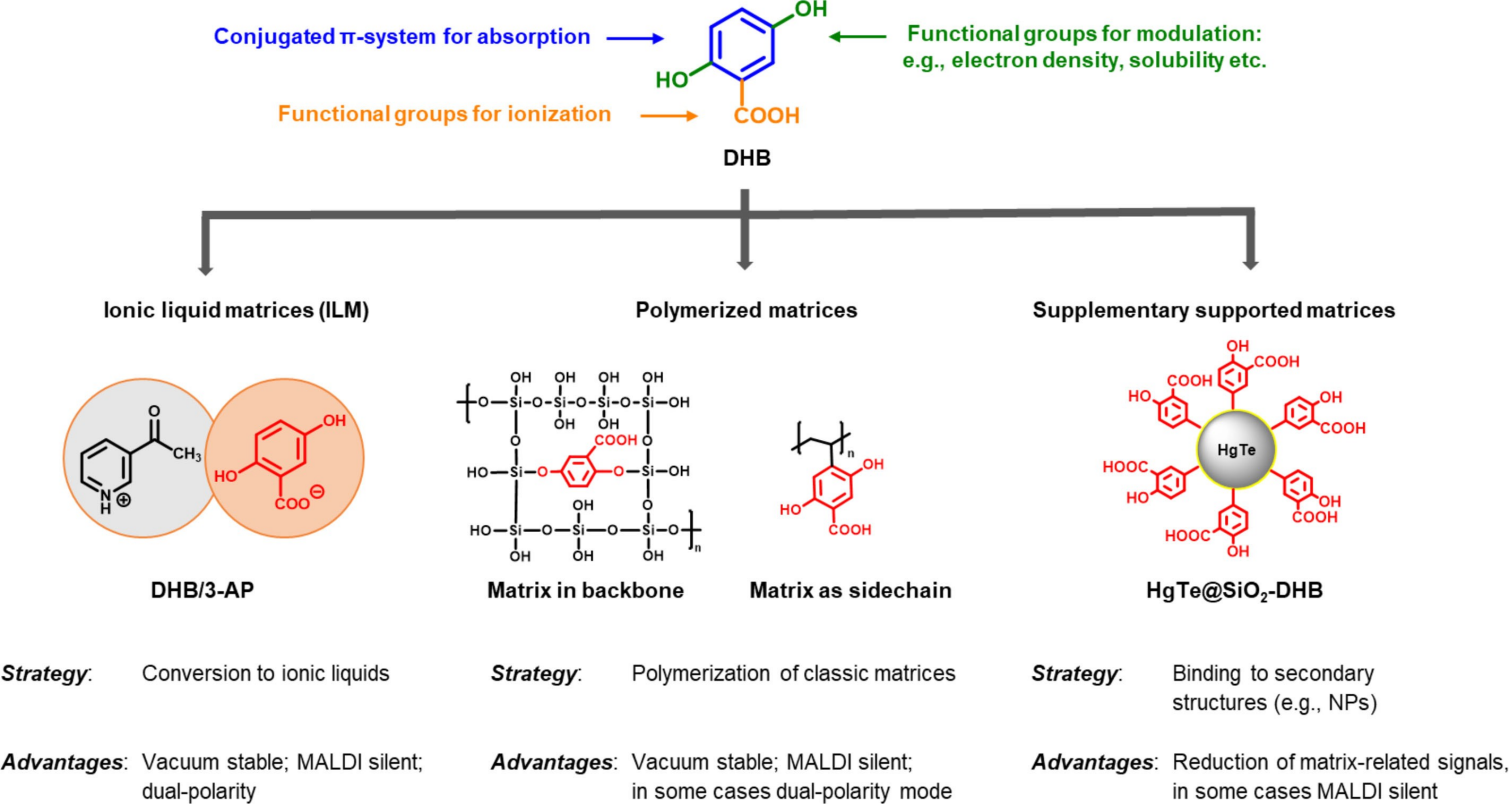
Rational design strategies and their corresponding advantages.[^41^, ^50^, ^52^, ^126^]

Many strategies aiming for vacuum‐stable and MALDI silent matrices seek to increase the molecular weight of the matrix (Figure 1), e. g., via polymerization (subchapter 3.1), binding to supplementary structures such as cyclodextrin (subchapter 3.1) or nanoparticles (subchapter 3.3), or the utilization of high molecular weight matrices such as conjugated oligomers or polymers (subchapter 3.2). As these matrices will generally not desorb, the ionization process is likely more comparable to surface‐assisted laser desorption/ionization mass spectrometry (SALDI‐MS). While the SALDI‐MS is not as deeply studied, several thermal and non‐thermal desorption and ionization processes are discussed, and a recent review gives an excellent summary.^[31]^

### 2.2 Strategies allowing to use classic matrices for LMW analytes

Several strategies were successfully implemented to minimize the background interferences of classic SOMs, which we will briefly summarize here, namely (i) pretreatments, (ii) instrumentation improvements, (iii) matrix suppression effect (MSE), and (iv) new deposition strategies.

An ideal sample pretreatment would remove all unwanted chemical species from the surface.^[32]^ For example, polyethyleneoxide (PEO)‐type surfactants, which can suppress the ionization of analytes due to their high ionization efficiency, can be removed by carefully washing with a methanol solution.^[33]^ Besides, hardware advances regarding the sensitivity, laser frequency and MS/MS capabilities brought many advantages such as high detection sensitivity, resolution accuracy, low sample consumption and rapid detection, which all significantly improve LMW compound analytics.[^16^, ^17^, ^34^, ^35^, ^36^]

Utilizing the matrix suppression effect (MSE) can provide high quality MALDI mass spectra (strong analyte signals with weak or none background interferences) and was first introduced by McCombie and Knochenmuss.^[37]^ The authors demonstrated that MSE can be achieved by adjusting factors such as laser intensity and the molar ratio between matrix and analyte in the sample. Also, novel deposition methods were reported for conventional matrices to produce a more homogeneous co‐crystallization between analyte and matrix,[^30^, ^38^] which strongly suppresses the so‐called coffee stain effect and decrease the time of measurements without searching for “sweet spots”.^[39]^

## 3 Rational design of newly developed matrix systems

### 3.1 Modifying well‐known matrices for LMW compound analytics

The structural modification of well‐known SOMs can make them vacuum stable and “MALDI silent” in the LMW region, thereby enabling the utilization for LMW compound analytics. Possible routes are (i) converting the SOM into ionic liquids, (ii) binding it to supplementary structures to increase the molecular weight, and (iii) polymerizing the SOM.

Ionic liquids (ILs) are liquid phase salts, i. e., salts which melt without decomposing.^[40]^ They have a number of interesting characteristics and potential applications, e. g., as electrolytes due to their inherent ionic conductivity. SOMs can be converted into ILs through acid/base couple combinations, yielding so‐called ionic liquid matrices (ILMs). The ionic structure renders the ILMs vacuum stable, and significantly reduces the matrix‐related signals as usually only the base component leads to a strong signal. Also, ILMs give homogeneous coatings, attractive for high throughput measurements as well as MSI. Conventional matrices such as DHB, CHCA or sinapinic acids (SA) were used as acidic partner, and ILMs generated by mixing with an organic base, e. g., amines, in equimolar ratio.^[43]^ Meriaux et al. synthesized ILMs by combining DHB with different nitrogen‐containing bases, i. e., 3‐acetylpyridine (3‐AP), aniline (ANI), and pyridine (Pyr) (i. e., DHB/3‐AP, DHB/ANI, and DHB/Pyr)^[41]^ for identification of lipid species with phosphocholine. The resulting ILMs provided a homogeneous matrix cover and supported MSI measurements more than 10 hours. Furthermore, some of the ILMs had dual polarity mode ability: e. g., DHB/3‐AP allowed the detection of lipids in both positive and negative mode. ILMs were successfully used to a variety of LMW molecules such as carbohydrates,^[44]^ oligosaccharides^[45]^ and lignin.^[46]^

Binding SOMs to a supplementary structure can increase the molecular weight of the matrix, and in some cases even enable noise‐free measurements in the LMW region. For instance, CHCA was covalently bound to different aromatic compounds via a Knoevenagel reaction to deliberately shift the molecular weight. While the matrix‐related background signals were moved to higher *m*/*z*, the derivatives maintained considerable chemical similarity to CHCA^[47]^ allowing to reliably detect certain classes of nucleotides and small pharmaceutical drugs. Cyclodextrin (CD) is a macrocycle capable of hosting smaller molecules in its cavity. The incorporation of an SOM in the macrocyclic cavity leads to a reduced observance of SOM related fragment ions and alkali metal ion adducts. E.g., the insertion of 2,4,6‐trihydroxyacetophenone (THAP) allowed the detection of testosterone and diazepam,^[48]^ and similarly, after incorporation of DHB in the CD cavity, a number of nonderivatized sex steroids, e. g., estrone, β‐estradiol, testosterone and progesteron were successfully analyzed.^[49]^ Combining classic organic matrices with e. g., nanoparticles also can be described as binding them to a supplementary structure. Such hybrid systems are further discussed in subchapter 3.3.

**Figure 2:**
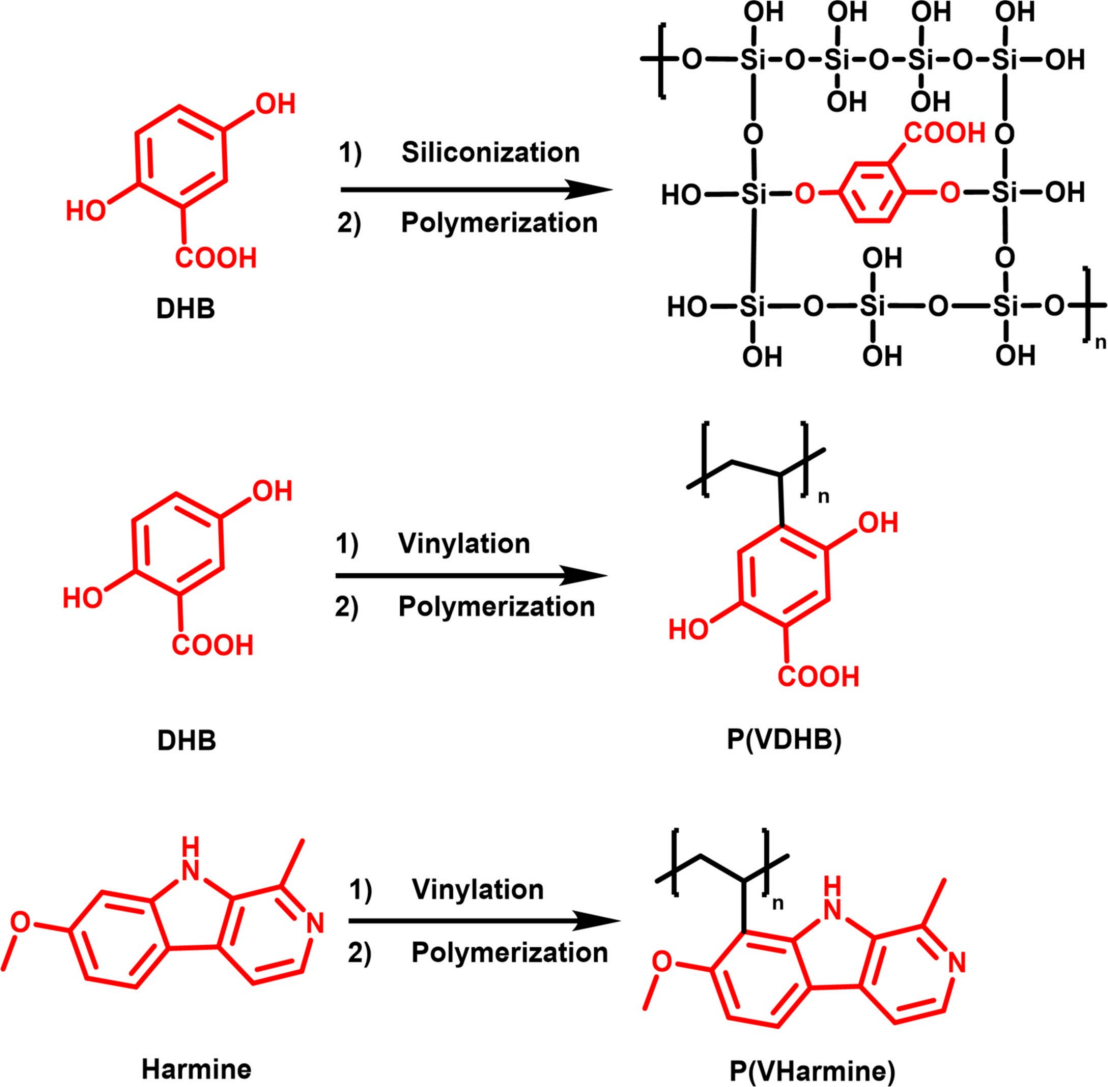
**Top**: Embedding DHB in a polysiloxane backbone to form a sol‐gel structure.^[50]^ **Middle**: Introducing vinyl groups to DHB to obtain polyethylene‐based P(VDHB) by radical polymerization. **Bottom**: Introducing vinyl groups to Harmine to obtain polyethylene‐based P(VHarmine) by radical polymerization.^[52]^

For polymerization, generally two different strategies are possible, i. e., incorporating the SOM in the polymer backbone, or binding it to the backbone as a sidechain. As the properties of the SOM (proton affinity, absorbance, …) should ideally be retained, the polymerization must not modify the chemical structure in a detrimental fashion, e. g., polymerization of DHB should not utilize the acidic function. Lin and Chen were the first to successfully incorporate a SOM into a polymeric structure^[50]^ (Figure 2). Hydrolyzation of tetraethoxysilane gave tetrahydroxylsilane, which was co‐condensed with DHB in a ratio of approximately 70 : 1 to yield a sol‐gel structure (Figure 2 **top**). In this structure, the SOM DHB is bound via its two hydroxy groups, and is an intrinsic part of the siloxane backbone (Si−O). As the carboxylic function is not touched, the matrix capability is mostly retained, while the embedment in the polysiloxane structure prevents the DHB from being desorbed as long as low laser intensities are being used. The polymerized SOM allowed to detect small biomolecules such as amino acids and small peptides, e. g., phenylalanine, arginin and bradykinin,^[50]^ while no significant DHB‐related signals were observed. Only when the amount of DHB in the sol‐gel structure was increased, or when higher laser intensities were needed, e. g., for measuring proteins, matrix‐related peaks were detected. In a similar fashion, CHCA was incorporated into a polydopamine structure:^[51]^ First, CHCA was reacted with a dopamine unit via carbodiimide coupling, then the resulting conjugate was co‐polymerized with an excess of dopamine. The resulting polymer was coated on a steel plate and SiO_2_ nanoparticles (making the system a hybrid matrix, see **3.3**) and UV/Vis spectra showed that the CHCA retained a strong band at ∼335 nm. Several LMW analytes, e. g., carbohydrates (glucose, sucrose) and amino acids (arginine, phenylalanine) were detected as cationic proton or sodium adducts. Only few CHCA‐related signals were detected, all with a very low intensity, making the system de‐facto MALDI‐silent.

SOMs can also be used as sidechains of polymeric backbones: Introducing vinyl groups to DHB and harmine allowed to obtain polyethylene‐based P(SOMs) by radical polymerization (Figure 2 **middle and bottom**). In this structural layout, the connection to the polymer backbone is achieved via a newly introduced carbon‐carbon bond, while the original functional groups of the matrix are not modified.

Consequently, the SOM sidechains retain their functionality and define the matrix performance, while the polyethylene backbone is chemically inert and imparts vacuum stability. The P(SOMs) have competitive performances regarding analyte scopes and ionization efficiencies when benchmarked against the corresponding SOMs. At the same time, the polymerization makes the P(SOMs) fully vacuum stable and MALDI silent. Also, the P(SOMs) are active in dual polarity mode and, when used in MALDI MSI, have the ability to produce and spatially resolve both positive and negative tissue‐related ions directly from cancer tissue.^[52]^

### 3.2 High‐molecular weight matrices

Classics SOMs can be modified to increase their molecular weight, potentially achieving vacuum stable and “MALDI silent” matrices. An alternative solution is to search for new matrices which already have a molecular weight outside the LMW region. We will focus on three classes, namely (i) porphyrin‐ and phthalocyanine‐based matrices, (ii) heteroaromatic oligomers and (iii) conjugated polymers.

Porphyrins as well as phthalocyanines are nitrogen‐containing conjugated macrocycles, which absorb strongly in the region of 400 nm. The molecular weight of the unsubstituted structures is 308.3 g/mol and 514.5 g/mol, respectively, but a wide range of substitution patterns are easily accessible. Substitution not only increases the molecular weight, but also allows to introduce basic or acidic functionalities at the peripheral sites. For non‐metalated derivatives, the lone pairs on the nitrogen atoms can be protonated by acid analytes. Ayorinde et al. were the first to use a porphyrin structure, namely *meso*‐tetrakis(pentafluorophenyl)‐porphyrin (F20TPP, MW 974.57) for the analyses of LMW nonylphenol ethoxylates (m/z range from 331–771 Da).^[53]^ The same authors also reported the analysis of fatty acids^[54]^ as well as sugars, ascorbic acid and other LMW analytes.^[55]^ F20TPP was also used for investigating pharmaceutical compounds, e. g., HIV protease inhibitors.^[56]^ Interestingly, F20TPP provides a higher cationization efficiency for small molecules when lithium or sodium salts are added,^[57]^ thus leading to a better resolution. Later on, 17 porphyrin matrices were tested by Chen et al.^[58]^ for the analyses of water‐soluble vitamins B1, B2, B6, B12 and C. It was shown that hydroxyl or carboxyl substituents are beneficial for vitamin analysis. While phthalocyanines most strongly absorb in the region of 700 nm, a second maximum is found in the region around 350–400 nm. A series of metal‐phthalocyanines (MPcs) with aromatic or aliphatic groups and different metal centers were tested as HMW matrices:^[59]^ Due to metal center, these compounds can form MPc‐analyte adducts, thereby shifting the analyte related signals to higher m/z regions, and enabled the detection of a variety of small molecules, including amino acids, peptides, and fatty acids.

Heteroaromatic oligomers generally show sufficient absorption at 337 nm. Depending on the number of units (and the substituents), the absorption maximum as well as the ionization potential can be shifted, which, together with well‐established substitution reactions, enables targeted structural modifications. Thiophene is an excellent example as a small heteroaromatic compound which can be modified at all four carbon positions and is easily oligo‐ and polymerized via the 2 and 5 positions. 2,3,4,5‐Tetrakis(3′,4′‐dihydroxylphenyl)‐thiophene (DHPT) was tested for the analysis of LMW compounds.^[60]^ Compared with CHCA, DHPT shows a much‐simplified mass spectrum in the LMW range, as only the DHPT radical cation (m/z 516.1) is observed in positive ion mode, while in negative mode the deprotonated molecule was detected at m/z 515.1. However, the authors point out that the analyte scope is limited; while a range of LMW compounds including glucose fatty acids and lipids were tested, only amines gave strong and reliable signals. This was explained by the Brønsted‐Lowry acid‐base theory: DHPT is a Brønsted‐Lowry acid and has high tendency to donate protons in the presence of Brønsted‐Lowry bases (e. g., amines).

Woldegiorgis et al. were the first to investigate oligothiophenes and oligobenzodioxins for LMW compound analytics.^[61]^ The analyte ions generated by these oligomers are radical cations obtained by charge transfer: the oligomers are electron‐transfer (ET) matrices. While the application of these matrices is restricted, and the molecule‐ions of the oligomers are clearly present, the versatility of the oligomer approach is clearly shown. Castellanos‐García et al.^[62]^ investigated oligo‐*p*‐phenylenevinylene (PV) derivatives as ET matrices for the analysis of LMW complexes and aromatic compounds. The PV derivatives form radical cations at low laser intensity, and the derivative with electron‐withdrawing CN substituents showed a particularly good performance as matrix. Similarly, *α*‐cyanophenylenevinylene derivatives can be used as ET matrices for LMW compounds such as triphenylamine dyes or pophyrine‐based biomarkers.^[63]^

Soltzberg and coworkers^[64]^ used a conjugated polymer as matrix for LMW compounds. Poly(3‐octylthiophene‐2,5‐diyl) absorbs strongly at 450 nm, and allowed to detect LMW acids in negative mode, e. g., *trans*‐cinnamic acid, salicylic acid and *p‐*aminobenzoic acid. The polymeric structure is a perfect example for an HMW matrix: While the absorption maximum of the conjugated polymer is at higher wavelengths, there is sufficient absorption at 337 nm. At the same time, the high molecular weight grants vacuum stability and interference‐free analysis of the LMW area. In organic electronics, conjugated polymers are routinely coated into thin films with surface roughnesses in the low nm range. Such uniform and defect‐free films are interesting for high‐resolution MSI.

**Figure 3:**
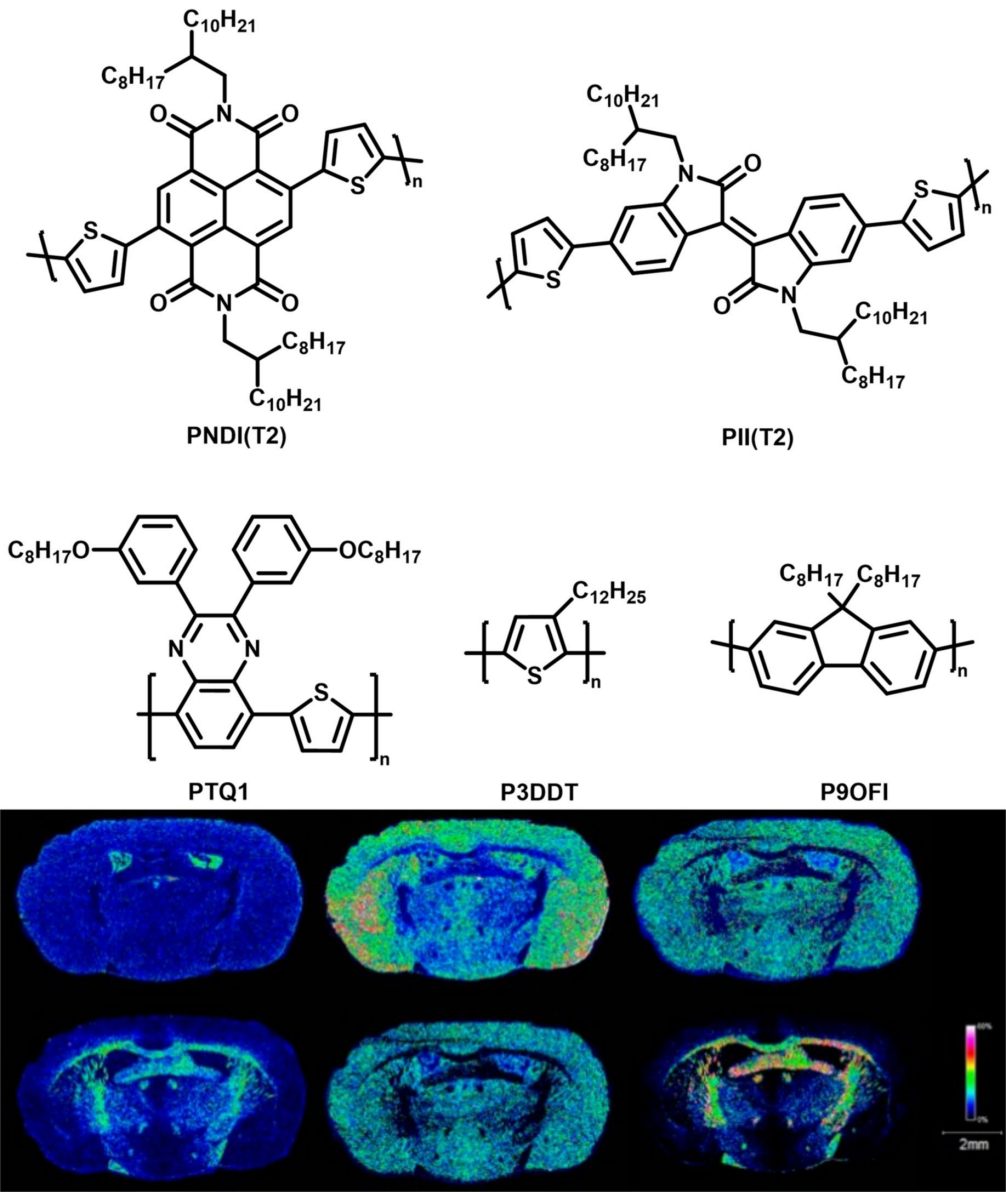
**Top**: molecular structures of conjugated polymer PNDI(T2), PII(T2), PTQ1, P3DDT and P9OFl^[19]^ **Bottom**: Ion images of selected mass channels acquired by MALDI MSI of a coronal rat brain section in negative mode and by using P3DDT as matrix. The distributions of the ionic species are visualized as heat maps. Ionic species (m/z) from top left to bottom right: 113.1 Da (±0.3 Da); 124.0 Da (±0.2 Da); 210.9 Da (±0.3 Da); 309.5 Da (±0.3 Da); 384.7 Da (±0.3 Da); 904.7 Da (±0.3 Da).^[19]^

Recently, we were able to show that other conjugated homopolymers and hetero‐co‐polymers are also promising matrices for LMW compound ‐analytics with MALDI MS and MSI.^[19]^ Five conjugated polymers, poly{[*N*,*N*′‐bis(2‐octyldodecyl)‐naphtalene‐1,4,5,8‐bis(dicarboximide)‐2,6‐diyl]‐*alt*‐5,5′(2,2′‐bithiophene)} (PNDI(T2)), poly(3‐dodecylthiophene‐2,5‐diyl) (P3DDT), poly{[2,3‐bis(3‐octyloxyphenyl)quinoxaline‐5,8‐diyl]‐*alt*‐(thiophene‐2,5‐diyl)} (PTQ1), poly{[*N*,*N*′‐bis(2‐octyldodecyl)‐isoindigo‐5,5′‐diyl]‐*alt*‐5,5′(2,2′‐bithiophene)} (PII(T2)), and poly(9,9‐di‐*n*‐octylfluorenyl‐2,7‐diyl) (P9OFl) were investigated as matrices (Figure 3 **top**)^[19]^ for the analyses of pharmaceutical compounds such as reserpine (RP), used for the treatment of high blood pressure, and the insecticide coumaphos (CP). The conjugated backbones absorbs strongly at the commonly used MALDI MS laser wavelength and together with the high molecular weight, make the polymers “MALDI silent”, enabling also efficient LMW compounds detection with MALDI MSI (Figure 3 **bottom**).^[19]^ When measuring the LMW spectra of a Gingko biloba extract, the polymers detected a comparable number of peaks as DHB in positive and 9‐aminoacridine (9AA) in negative mode, making them competitive dual polarity modemode matrices.

The utilization of conjugated polymers is also interesting from a mechanistic point of view: The analyte is often described as being a defect in the crystal lattice of the matrix. Conjugated polymers are usually semicrystalline, i. e., contain amorphous and crystalline regions. By changing the sidechain length and type, we could obtain semicrystalline and fully amorphous derivatives of PNDI. The latter not only functions as matrix for LMW compounds, but even slightly outperforms the former, suggesting that for conjugated polymers the analyte incorporation most likely takes place in the amorphous phase.^[65]^

### 3.3 Binary, hybrid and nanomaterial‐based matrices

Other strategies seek to exploit synergistic effects obtained by combining different components into one matrix system. Such multicomponent systems are (i) binary matrices (consisting of two conventional organic MALDI matrices) and (ii) hybrid matrices (consisting of an organic and an inorganic compound), which are often based on (iii) inorganic matrices.

Solouki et al. first used the binary matrix systems 4‐nitroaniline/coumarin and fructose/DHB to improve the ionization efficiency of biomolecules such as bovine insulin and lysozyme, and to obtain a to better mass measurement accuracy.^[66]^ Guo and He combined two classic matrices with different proton affinities (PAs), 9AA and CHCA, achieving a significant reduction of matrix‐related signals in both positive and negative mode, potentially due to a proton transfer from the lower PA matrix to the higher PA matrix, and the suppression of other matrix‐related signals.^[67]^ Among other binary matrices for the analyses of LMW compounds are 3‐hydroxycoumarin (3‐HC) and 6‐aza‐2‐thiothymine (ATT), which were used for the detection of drugs and single amino acids.^[68]^ A 1,8‐bis(dimethylamino)naphthalene (DMAN)/9AA binary matrix was deployed for the analysis of a number of compound classes, e. g., free fatty acids, mono‐, di‐ and tri‐glycerides, phospholipids, glycolipids and cardiolipins.^[69]^ Blending *N*‐butyl‐4‐hydroxy‐1,8‐naphthalimide (BHN) and DHB allowed the measurement of *O‐*acetyl‐L‐carnitine hydrochloride, oxytocin, and small peptides.^[70]^

Combining organic and inorganic components, such as classic matrices and nanoparticles, to a hybrid matrix was first considered by Lin et al. to detect LMW analytes.^[71]^ Commonly used matrices such as DHB and CHCA can conjugate onto functionalized magnetic nanoparticles (MNPs), enabling a background‐free detection in MALDI MS as the SOM is tied to and stabilized by the MNP surface. In this regard, the approach bears similarity to the strategies discussed under subchapter **3.1**. A hybrid system consisting of silicon nanoparticles and DHB/CHCA was used to analyse different LMW compounds such as inorganic light‐emitting molecules, di‐ and triacylglycerols and cholesterol esters, solar cell materials, and dendrimers;^[42]^ Polydopamine (PDA)‐capped AgNPs (AgNPs@PDA) were used to analyse glycerophospholipids and sphingolipids in both positive and negative ion modes (dual polarity mode ability, see subchapter **3.5**).^[72]^

Inorganic carbon‐, silicon‐ or metal‐based materials, such as graphene and metal‐organic frameworks (MOFs), have a high surface area and are vacuum stable. Their utilization is usually classified as surface‐assisted laser desorption/ionization mass spectrometry (SALDI‐MS), and several excellent publications give further details.[^31^, ^73^, ^74^, ^75^, ^76^] Therefore, we will only highlight some selected examples: Dong et al.^[77]^ used graphene for the analyses of amino acids, anticancer drugs, nucleosides, and steroids. Due to the high surface area, graphene can efficiently capture analyte molecules. The single‐layer morphology leads to better energy transfer and minimized background signals, providing a high sensitivity. Graphene‐based materials were also employed for analyses of pollutants like nitropolycyclic aromatic hydrocarbons^[78]^ as well as small peptides, lipids, and glycosylated metabolites.^[79]^ MOF‐ based matrices were utilized by Chen et al.^[80]^ to detect small molecules such as amino acids, nucleosides and alkaline drugs. MOFs both strongly adsorb analyte molecules and efficiently transfer energy onto them, enabling low background interferences and high desorption/ionization efficiencies. Inorganic and inorganic/organic hybrid materials are especially interesting for “lab‐on‐a‐chip” LDI‐MS solutions for clinical research, e. g., to enable metabolic analyses.[^74^, ^81^, ^82^, ^83^]

### 3.4 Reactive matrices

Reactive matrices act not only as matrices for the MALDI MS process, but also as derivatization agents which selectively react with certain functional groups of the analytes. The resulting derivatization products can display more efficient ionization, higher mass resolution, or an advantageous shift of the molecular mass. The utilization of chemical derivatization agents followed by the application of a conventional matrix, e. g., CHCA or DHB, has also been classified as reactive matrix,[^84^, ^85^, ^86^, ^87^] and there is an excellent recent review discussing on‐tissue chemical derivatization (OTCT) reagents for MALDI MSI.^[84]^ Here, we will focus on reactive matrices in a narrow sense, i. e., organic compounds with derivatizing as well as matrix functionality, which enable detection of LMW compounds containing (i) carbonyl functions, (ii) amino‐ and hydroxy groups, and (iii) C−C double bonds.

Carbonyl groups such as aldehydes and ketones react with amines to form the corresponding Schiff base. Zhang and Gross^[88]^ first reported the utilization of anthranilic acid (AA) as reactive matrix to exploit this reactivity for the analysis of oligodeoxynucleotides (ODNs): The amino group of AA can react with the open‐chain aldehyde function at an ODN's abasic site. The resulting Schiff bases show intense signals with a 119 Da increase in mass, and furthermore a series of defined cleavages is promoted, allowing to identify and locate abasic sites in model ODNs. 2‐Phenyl‐3‐(*p*‐aminophenyl) acrylonitrile (PAPAN) is an amino‐containing *α*‐cyanocinnamic acid (CCA) derivative similarly capable of forming Schiff bases with carbonyl groups. Combining this reactivity with CCA's high UV absorption efficiency improved the detection sensitivity 100‐fold compared with using DHB, while the induced fragmentation reactions for glycans could facilitate the glycan structure interpretation.^[89]^

Hydrazines condense with carbonyl groups to form hydrazones, a reaction historically used for the characterization of carbohydrates, e. g., by Emil Fischer. 2,4‐Dinitrophenylhydrazine (DNPH) was introduced by the group of Schiller^[90]^ as a reactive matrix to analyse native and oxidized lipids with aldehyde groups. DNHP converts the aldehydes into the corresponding hydrazones, which are easily identified, lowering the detection limit and giving mass spectra with low background noise. Several other hydrazide‐ or hydrazine‐containing compounds were also reported as reactive matrices for gaseous aldehydes containing carbonyl groups (e. g., formaldehyde, acetaldehyde and propionaldehyde)^[91]^ as well as for ketones (e. g., acetone, methyl ethyl ketone, and methyl isobutyl ketone).^[92]^

**Figure 4:**
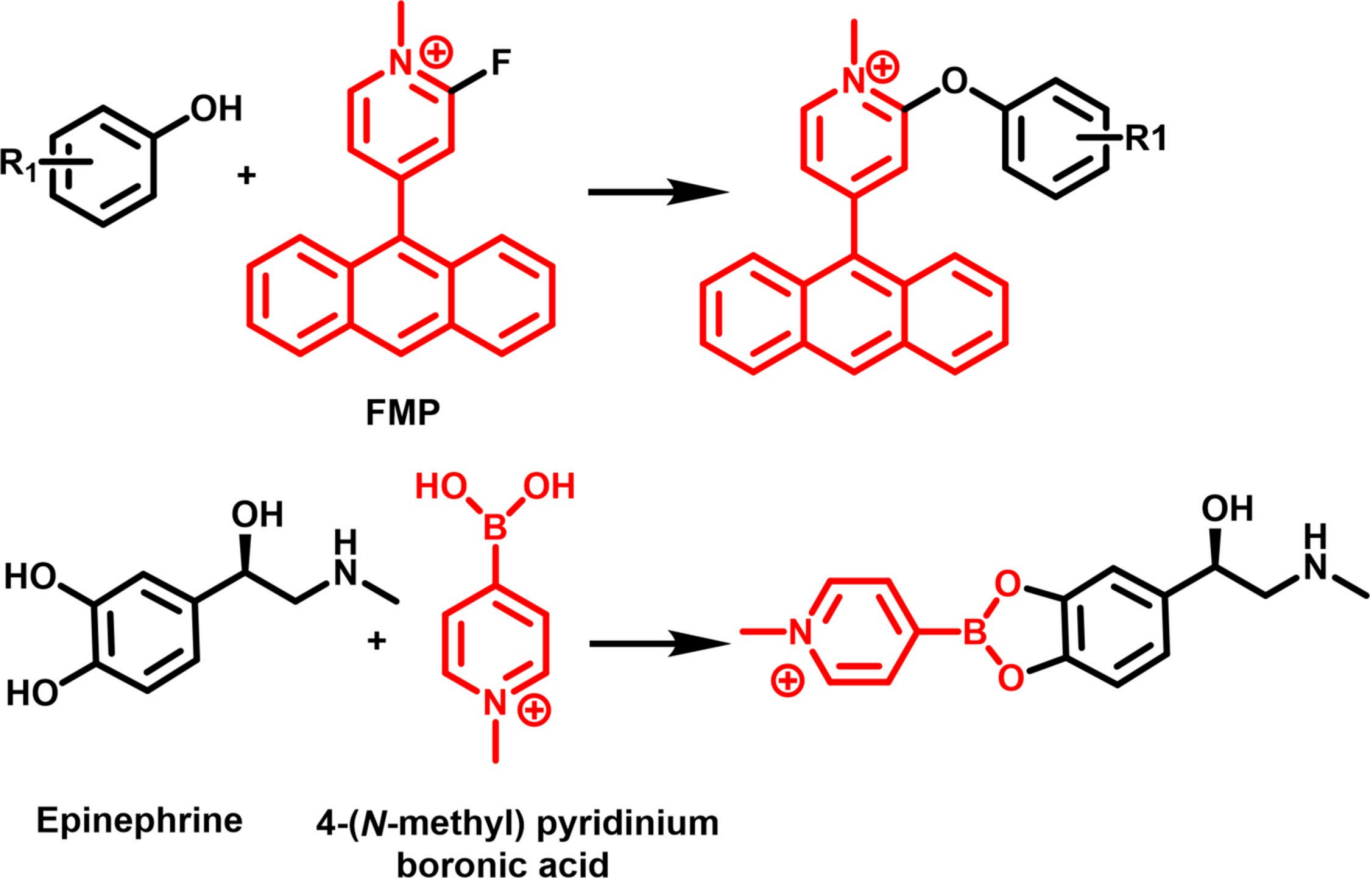
**Top**: FMP as a reactive matrix to detect analytes with hydroxy groups.^[97]^ **Bottom**: Derivatization of epinephrine with the reactive matrix 4‐(*N*‐methyl)pyridinium boronic acid.^[98]^

Analytes carrying amino functions can be targeted by pyrylium cations, six‐membered aromatic heterocycles containing a positively charged oxygen atom. Pyrylium reacts with primary amines to yield the corresponding pyridinium cations, a conversion which often takes place already at room temperature under mild conditions. 2,4‐Diphenyl‐pyranylium (DPP) was first tested as reactive matrix to target dopamine (DA), a neuroactive substance possessing a primary amine functional group (Figure 4), and allowed to map the distribution of DA in brain tissue sections.^[93]^ Pyrilium derivatives were since used in a number of procedures, yet sometimes required an additional matrix to obtain high enough ionization efficiencies.^[94]^ The aforementioned Schiff‐base formation can also be used for amine‐containing analytes: Some aromatic carbonyl compounds, for instance 4‐hydroxy‐3‐methoxycinnamaldehyde (CA), 2,4‐dihydroxybenzaldehyde (DHBA) and 2,5‐dihydroxyacetophenone (DHAP) were reported as reactive matrices for the analysis of amines.[^95^, ^96^] Recently, 4‐(anthracen‐9‐yl)‐2‐fluoro‐1‐alkylpyridines (FMP) were used to detect neurotransmitters (NTs) containing amino‐ and hydroxy groups.^[97]^ FMP consist of two domains: the fluoropyridine moiety reacts with primary and secondary amines as well as hydroxy groups via a nucleophilic aromatic substitution, while the extended conjugated system of the anthracene unit sustains a strong absorption.

**Figure 5:**
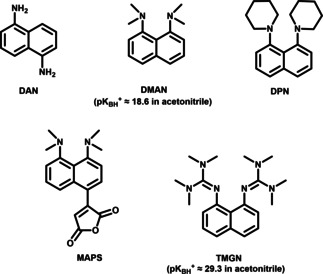
Structures of **DAN** and some archetypal proton sponge matrices. **DAN**: 1,5‐Diaminonaphthalene;^[124]^ **DMAN**: 1,8‐Bis (dimethylamino) naphthalene;^[69]^ **DPN**: 1,8‐Di(piperidinyl)naphthalene;^[121]^ **MAPS**: 3‐(4,5‐Bis (dimethylamino)napthalen‐1‐yl)furan‐2,5‐dione;^[122]^ **TMGN**: 1,8‐Bis(tetramethyl‐guanidino)naphthalene.^[123]^

When they are reacted with boronic acids, 1,2‐ and 1,3‐diols form stable cyclic boronated esters (Figure 4). 4‐(*N*‐Methyl) pyridinium boronic acid was used to selectively derivatize catecholamines and to sensitively detect them:^[98]^ The boronic acid function of the reactive matrix condenses with the diol functionality of catechol, and the permanent charge of the pyridinium increased the ionization efficiency of the targeted analytes. The C−C double bond of olefins can react with carbonyls through the Paternò‐Büchi (PB) reaction, a 2+2 photocycloaddition yielding oxetanes, four‐membered heterocycles. Benzophenone (BPh) derivatives undergo PB reactions and at the same time show good absorption behavior as needed for the MALDI MS process. This was used by the group of Heiles for the on‐tissue derivatization and detection of unsaturated phospholipids (PLs)^[99]^ and allowed to visualize 12 lipid classes from mouse kidney and pancreas tissue through MALDI MSI.^[100]^

### 3.5 Negative and dual polarity mode matrices for LMW compounds

Most LMW compound analytics focuses on positive ion mode, yet negative ion mode spectra often have the advantage of lower background signals and higher reproducibility. Here, we will briefly discuss examples of two matrix classes enabling negative or dual polarity mode (i. e., negative and positive mode) measurements, (i) proton transfer matrices (ii) electron transfer matrices.

While MALDI matrices for positive ion mode often carry acidic functional groups, e. g., DHB and CHCA, proton transfer‐based matrices for negative mode have to be moderately strong bases. In line with the Brønsted‐Lowry acid‐base theory, such matrices can abstract protons from the analyte. Consequently, most proton transfer matrices for negative mode measurements contain amino functions.

The amino group of 9AA accepts protons form acidic analytes, leading to the formation of deprotonated and negatively charged analyte species[A−H]^−^. When 9AA was introduced as MALDI matrix, a number of LMW analytes such as phenols, carboxylic acids, sulfonates, amines and alcohols were successfully detected.^[101]^ Later on, the analyte range was widened to e. g., organic acids,^[102]^ oligosaccharides,^[103]^ phospholipids^[104]^ and metabolites.^[105]^ 2‐Aminoacridine (2AA),^[106]^ an isomer of 9AA, was also used to detect resins and real‐life varnish samples.

4‐Phenyl‐*α*‐cyanocinnamic acid amide (Ph−CCA−NH2) was synthesized by converting the acidic carboxyl group of CHCA in an amide group. This allowed improved sensitivity and reproducibility lipid measurements in negative mode and suppressed matrix related signals.^[107]^ 2‐(2‐Aminoethylamino)‐5‐nitropyridine (AAN),^[108]^ quercetin^[109]^ and norharmane (NOR)^[44]^ have also been described for negative ion MALDI MS analysis of different lipid classes. Other examples for proton‐accepting matrices are *N*‐(1‐naphthyl) ethylenediamine dinitrate (NEDN) and 1‐naphthylhydrazine hydrochloride, which were used to detect oligosaccharides, peptides, metabolites and explosives in negative ion mode.^[110]^ Recently *N*‐(1‐naphthyl) ethylenediamine dihydrochloride (NEDC) and 1,8‐bis(pyrolidinyl)naphthalene (BYPN) were also used to map metabolite distributions in mouse eyes.^[96]^

Another strong amino‐containing base, 1,5‐diaminonaphthalene (DAN), was first reported for negative mode MALDI MS measurements of gangliosides by Juhasz and coworkers.^[111]^ Afterwards DAN was reported for dual polaritymode analyses of LMW phospholipids (PL) (m/z<1000),^[112]^ and DAN hydrochloride salt permitted to visualize the distribution of small metabolites such as metal ions, amino acids, carboxylic acids, nucleotide derivatives and lipids through MALDI MSI of liver, brain, and kidney tissues from mice with high sensitivity.^[113]^ Also, DAN hydrochloride performs well for the detection of neutral molecules such as saccharides, which are difficult to ionize: Saccharides can form ion adducts with the Cl^−^ and are easily ionized by using DAN. Recently, flavonoids such as flavan‐3‐ols, procyanidins in peanuts or strawberries were identified, and it was demonstrated that flavan‐3‐ols prefer to form the deprotonated molecule [M−H]^−[114,115]^.

A group of negative ion mode matrix are so called “proton sponge” derivatives. “Proton sponge” is the trade name of 1,8‐bis(dimethylamino)naphthalene (DMAN), a diamine structure in which the two dimethylamino groups are attached on the same side. Compared with classic MALDI matrices, DMAN is a very strong base (p*K*_BH_+≈18.6 in acetonitrile) and is able to deprotonate acidic analytes in the liquid‐phase to form an ion pair [DMAN+H]^+^/[M−H]^−^. At the same time, the naphthalene core is favorable for UV absorption. As DMAN generates no matrix‐related peaks in the spectrum and yields a very clear signal of the deprotonated analyte, it is widely used to detect fatty acids, amino acids, fatty acid‐amino acid conjugates, plant and animal hormones, vitamins, and short peptides.^[117]^ Also it was used for the detection of saturated, unsaturated and dicarboxylic acids with high S/N ratio and without interference from matrix‐related fragment ions.^[118]^

Several superbasic 1,8‐bisphosphazenyl‐naphthalene proton sponges were synthesized by Kögel et al..^[119]^ Here, the methyl substituents found in the dimethylamino groups of DMAN are replaced by *p‐*alkyl functions. Through introducing diverse *p*‐amino and *p*‐alkyl groups as *N*‐substituents, a significant increase in basicity was obtained. These proton sponges have p*K*_BH_+ values between 30 to 32 and can detect hard ionizable small compounds such as steroids, sterols, fatty alcohols, and neutral saccharides.^[120]^ Later on, other bisphosphazene proton sponges were designed to further increase the ionization ability and vacuum stability. In 1,8‐di(piperidinyl)naphthalene (DPN) 1,8‐dinitrogen center is surrounded by two piperidinyl groups. These hydrophobic and bulky groups improve the ion‐pair change separation, leading to increasing of signal intensity, and after formation of the[DPN+H]^+^ adduct, prevent the protons from dissociating again, resulting in an improved ionization.^[121]^ Also, compared with DMAN, DPN has higher vapor pressure because of its higher molecular weight, and in an MSI experiment taking 7 hours, ion maps of myristoleic acid ([M−H]^−^ m/z 225), myristic acid ([M−H]^−^ m/z 227), palmitic acid ([M−H]^−^ m/z 255), oleic acid ([M−H]^−^ m/z 281) were acquired in negative ion mode. Another example aimed to achieve higher molecular weight to increase the vacuum stability is 3‐(4,5‐bis(dimethylamino)napthalen‐1‐yl)furan‐2,5‐dione (MAPS):^[122]^ A maleic anhydride is added in the para position of DMAN through a Tyler reaction, yielding a vacuum stable derivative. Furthermore, MAPS is the first matrix which allowed a reliable LMW compounds detection in the area under 200 m/z (e. g., of chloride at m/z 35 Da). Water‐soluble 1,8‐bis(tetramethylguanidino)‐naphthalene (TMGN) was reported as matrix for the quantitative detection of perfluorinated compounds (PFCs) in environmental water.^[123]^

Proton transfer‐based matrices are usually applied to detect analytes with relatively high ionization potentials. For analytes with low ionization potentials, the electron transfer (ET) mechanism plays a vital role. The charged analyte molecules generated by ET matrices are radical ions obtained by charge transfer.

Conjugated oligomers can function as ET matrices, e. g., the oligothiophenes and oligobenzodioxins introduced in subchapter **3.2** yield radical cations for positive ion measurements of LMW compounds.[^62^, ^63^] Similarly, the di‐heterocycle tetrathiafulvalene (TTF) was reported as ET matrix for negative mode measurements by Asakawa et al:^[125]^ Oxidation, i. e., the transfer of electrons onto suitable analyte molecules, converts each ring to a stable aromatic 6π‐electron system. TTF was used to detect several industrial pigments, and due to the high election donating ability of TTF, analytes were detected as radical anions instead of deprotonated ions [M−H]^−^.

Also, the conjugated polymers discussed under subchapter **3.2** are excellent dual polarity mode matrices, enabling LMW compounds detection in positive as well as negative mode, while being vacuum stable and MALDI silent, i. e., not giving matrix‐related signals when low laser intensities were used.[^19^, ^65^]

## 4 Summary and outlook

In recent years there is an increased interest to use MALDI also for LMW analytics, especially because of the potential of MALDI MS imaging. However, the application in LMW analytics is significantly restricted by the matrix‐related spectral interferences of traditional matrix systems. In this review, we summarized the research progress regarding the design and applications of matrices suitable for the MALDI MS analysis of LMW compounds, focusing on five areas: (i) The adaption of classic matrices for LMW analytics through structural modification. Polymerization or conversion into ionic liquids can make classic SOMs vacuum stable and “MALDI silent” in the LMW region; (ii) New high molecular weight matrices can be intrinsically vacuum stable, and potentially are “MALDI silent”. Here, conjugated polymers are a highly promising matrix class with outstanding properties combining suitable optical absorption and high ionization efficiency in dual polarity mode with being “MALDI silent” and vacuum stable; (iii) Binary and hybrid matrices, of which the latter have high promise for “lab‐on‐a‐chip” applications; (iv) Reactive matrices, which not only promote desorption and ionization of the analyte, but also derivatize it to allow advanced characterization; and (v) matrices which enable the analysis of LMW compounds in negative ion mode or even dual polarity mode.

When it comes to LMW analytes, there is no archetypical molecular target, instead, a multitude of different compound classes with different (physico‐)chemical properties require tailor‐made matrix systems. Polymerization can be a powerful tool to bestow vacuum stability onto a given high‐performance matrix and make the matrix “MALDI‐silent” in the LMW area. Such a strategy might even work for reactive matrices but this requires to carefully choose where to place weak bonds as predetermined breaking points. When developing matrices for LMW analytics, most strategies focus on increasing the molecular weight of the matrix, e. g., by polymerization or by binding to a supplementary structure. As such matrices are not or only fractionally vaporized, the desorption and ionization mechanisms are still unclear, and might be more closely related to SALDI processes.

Dual polarity mode matrices can enable a sensitive non‐targeted screening of the LMW area. Electron transfer (ET) matrices are among the most promising classes to achieve this, and when comparing the structures of the ET matrices, the overlap with the area of organic and polymer electronics is obvious. Here, inspiration might be taken from the conscious tuning of energy levels to achieve certain ionization energies.

The field of organic electronics can also be inspiring in a different way: For high‐resolution MSI, homogenous matrix layers are needed to ensure pot‐to‐spot reproducibility and allow analyte quantitation. The fabrication of homogenous and defect‐free films with roughnesses in the nanometer range is an obligate prerequisite to obtain high charge mobility, and a multitude of strategies and techniques were established to achieve this. A transfer to the area of MSI might be possible.

## Conflict of interest

The authors declare no conflict of interest.

## Biographical Information

*Zhi Qiao received his M.Sc. degree in 2018 from the TU Dresden. In 2019, he joined the Leibniz Institute of Polymer Research being enrolled as a Ph.D. student at TU Dresden under the supervision of Franziska Lissel and Brigitte Voit. His research interests mainly focus on the design and development of novel matrices for MALDI measurements, in particular for LMW compounds. He is supported by the China Scholarship Council*.

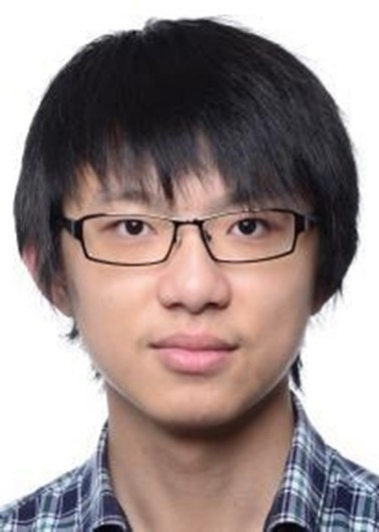

## Biographical Information

*Franziska Lissel is an Acting Professor at the University of Jena and a TU Dresden Young Investigator. As a Liebig Fellow of the FCI, she heads the Functional Electronic Materials Group at the Leibniz Institute of Polymer Research. Her group investigates the covalent introduction of redox‐active metalcenters into conjugated polymer backbones, develops polymeric MALDI matrices, new concepts for stretchable polymer electronics, and molecular switches. She was a postdoc at Stanford with Prof. Zhenan Bao and obtained her Ph.D. at the University of Zurich*.